# Polysomnography Differences Between Sleepy and Non-Sleepy Obstructive Sleep Apnea (OSA) Patients

**DOI:** 10.3390/healthcare13050478

**Published:** 2025-02-22

**Authors:** Izolde Bouloukaki, Theofilos Vouis, Antonios Velidakis, Violeta Moniaki, Eleni Mavroudi, George Stathakis, Michail Fanaridis, Sophia Schiza

**Affiliations:** Department of Sleep Disorders Center, Department of Respiratory Medicine, Medical School, University of Crete, 71500 Heraklion, Greece; theofvouis@gmail.com (T.V.); vmoniaki@yahoo.gr (V.M.); elenima23@hotmail.com (E.M.); stathakisgg@gmail.com (G.S.); schizas@uoc.gr (S.S.)

**Keywords:** obstructive sleep apnea, excessive daytime sleepiness, sleep architecture, indices of OSA severity, polysomnography, sleepy phenotype

## Abstract

**Background/Objectives:** Factors underlying excessive daytime sleepiness (EDS) in obstructive sleep apnea (OSA) are not fully understood. We investigated whether polysomnography (PSG) parameters differed between non-sleepy and sleepy (based on the Epworth Sleepiness Scale (ESS)) OSA patients with the same disease severity, which may play a role in the presence of EDS. **Methods:** A total of 1307 patients, without cardiovascular, metabolic, respiratory, or inflammatory comorbidities, diagnosed with OSA (apnea–hypopnea index (AHI) ≥ 5 per hour of sleep) with type 1 PSG were included. Based on the AHI, patients were classified into mild- (AHI 5–14.9, n = 236), moderate- (AHI 15–29.9, n = 367), and severe-OSA (AHI ≥ 30, n = 704) groups. These groups were further divided into two subgroups based on the ESS, the most convenient and widely used tool to assess excessive daytime sleepiness: sleepy (ESS > 10) and non-sleepy (ESS ≤ 10). PSG data were compared between groups, and multivariable logistic regression was used to identify differences after adjustment for confounders. **Results:** For the entire population, male sex, younger age, obesity, depression, increased wakefulness after sleep onset (WASO), the arousal index, shorter sleep latency, and all indices of OSA severity (AHI, oxygen desaturation index, mean and lowest resting room air pulse oximetry (SpO_2_), and sleep time with oxygen saturation < 90% (TST90)) were significantly associated with EDS. The arousal index consistently showed a strong association with EDS across all OSA severity groups. Moderate-OSA sleepy patients were younger, with shorter sleep latency and increased indices of OSA severity, excluding the AHI. Severe-OSA sleepy patients were younger, males, and obese; had depression, decreased slow-wave sleep (SWS) and sleep latency, and increased WASO; and presented an increase in all indices of OSA severity. **Conclusions:** Our results suggest that male sex, younger age, obesity, the presence of depression, WASO, lower sleep efficiency, the arousal index, and all indices of OSA severity may account for the presence or absence of EDS in OSA patients and could be useful for exploring the underlying pathophysiological mechanisms for precision medicine.

## 1. Introduction

Obstructive sleep apnea (OSA) is a multifactorial disease characterized by recurrent episodes of upper-airway obstruction during sleep, leading to apnea or hypopnea (complete or partial cessation of airflow for 10 s or more during sleep), ultimately resulting in intermittent hypoxia (periodic alternating exposures to hypoxia and normoxia), disrupted sleep architecture (the structured organization of the various sleep stages), and intrathoracic pressure swings [1]. The presence of OSA poses a significant challenge for physicians and healthcare systems globally due to its increasing prevalence, ranging from 9% to 38% [2], and associated mental and medical comorbidities [3,4]. Conventionally, the assessment of OSA severity is based on the apnea–hypopnea index (AHI), the combined average number of apnea and hypopnea events that occur per hour of sleep [5]. To meet the diagnostic criteria for OSA, a person must have either more than 15 respiratory events per hour or more than 5 events per hour along with typical symptoms of OSA, such as snoring, fatigue, excessive daytime sleepiness (EDS), or comorbid conditions like hypertension, coronary artery disease, or stroke [1,6,7]. However, nowadays, diagnostic polysomnography (PSG), a multi-parameter type of sleep study used to diagnose sleep disorders, contains a plethora of information that can be used for a more comprehensive analysis of OSA, moving beyond the basic frequency-based measures like the AHI. This encompasses parameters such as ventilatory, hypoxic, and arousal burden; ventilatory patterns; and Pulse Wave Amplitude Drops (sudden drops in pulse wave amplitude that reflect peripheral vasoconstriction resulting from sympathetic activation) [8].

OSA is considered a heterogeneous disease, characterized by diverse symptoms, anthropometric features, polysomnographic findings, long-term outcomes, and comorbidities [9]. The symptoms of OSA can vary widely, and they may not necessarily reflect the severity of the disease [10]. The presence of EDS is common among patients with OSA, affecting 40.5–58% at initial diagnosis, and is recognized as a primary symptom of the syndrome [11]. If it is present, it can result in diminished quality of life and increased societal burden, which may impact healthcare utilization and costs. More specifically, patients with OSA and EDS are more prone to reporting lower mental well-being, impaired cognitive abilities, decreased productivity, and increased rates of work and car accidents [11,12,13,14].

The factors underlying EDS in OSA are not fully understood. Existing research suggests a possible relationship between polysomnographic measures (sleep architecture and OSA severity parameters) and EDS in these patients, but findings have been inconsistent. More specifically, several studies have revealed a lack of strong or consistent association among the indices of OSA severity, such as the AHI, and subjective EDS [15,16,17,18]. The presence of EDS has been also attributed to chronic intermittent hypoxia and sleep fragmentation [19]. OSA patients with EDS often exhibit alterations in macrostructural characteristics, including increased sleep efficiency, reduced sleep latency, a greater proportion of NREM, stage N1 sleep, and a decline in slow-wave sleep (SWS), as well as alterations in the microarchitecture, including an elevated arousal index, compared with those without EDS [18,20,21,22]. However, research findings on these associations have yielded mixed results [16,23,24]. The variability in findings may be attributed to discrepancies in the definition and measurement of EDS across studies or to methodological variations in the evaluation of sleep quality and architecture, which are susceptible to experimental and technical influences.

Given the serious public health implications of EDS and its medicolegal aspects [25], understanding the determinant of this OSA phenotype is an area of important future research. Such insights may be gained by investigating the association between the sleepy phenotype, OSA severity indices, and sleep architecture parameters. Exploring the sleepy phenotype within groups with different OSA severity could also yield valuable findings. Therefore, the aim of our study was to investigate whether PSG parameters differed between non-sleepy and sleepy OSA patients with the same OSA disease severity at the time of diagnosis, in a large cohort of newly diagnosed OSA patients, without comorbidities and before treatment initiation. We hypothesized that identifying the PSG characteristics of the patients with sleepiness is an essential step in elucidating the link between OSA pathophysiology and the EDS experienced by these patients.

## 2. Materials and Methods

### 2.1. Design and Sample

A cross-sectional study of individuals visiting the University of Crete’s Sleep Disorders Center (Department of Respiratory Medicine, School of Medicine) in Greece for assessment of suspected OSA was conducted over an eight-year period (2015–2023). The inclusion criteria were (1) above 18 years of age, (2) with OSA diagnosis according to standard criteria (AHI ≥ 5 on PSG), and (3) treatment-naïve OSA.

Participants were excluded if they refused participation; had central sleep apnea syndrome, restrictive ventilatory syndrome, or any cardiovascular (coronary disease, atrial fibrillation, Cerebro-Vascular Accident/Transient Ischemic Attack, and/or heart failure), metabolic (except for hyperlipidemia), respiratory, or inflammatory comorbidities; had a personal or family history of mental illness, intake of benzodiazepines or similar sleep-inducing drugs, drug or alcohol abuse, or severe cognitive impairment; or had a history of narcolepsy or restless legs syndrome. Ethical approval (number 7370/04-06-2014) was granted by the University Hospital Ethics Committee, and all participants provided written informed consent.

**Figure 1:**
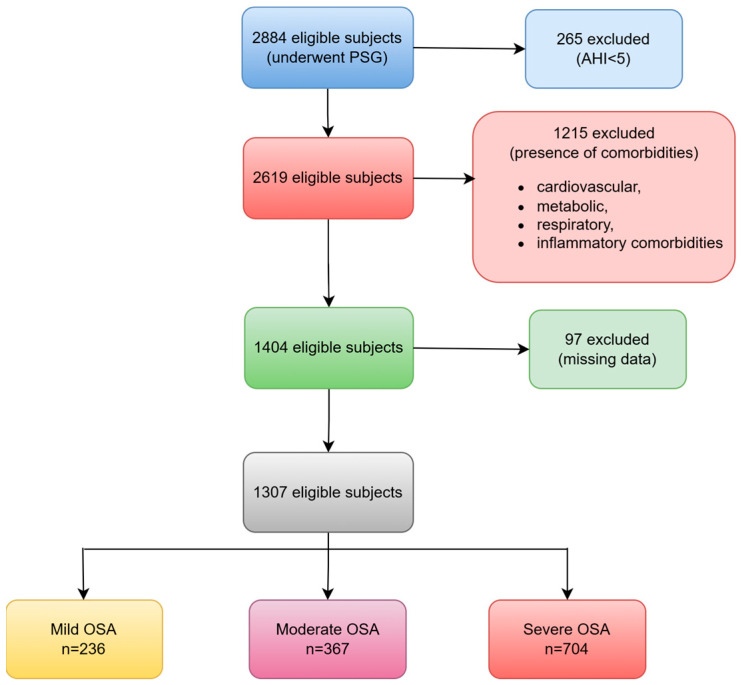
The flowchart of patients finally included. PSG: polysomnography; AHI: apnea–hypopnea index; OSA: obstructive sleep apnea.

Out of the 2884 adults who were assessed for suspected OSA, 2619 of them (91%) received a confirmed diagnosis of OSA. Following the exclusion of patients with specific comorbidities and missing data, our final sample comprised 1307 patients (Figure 1).

### 2.2. Data Collection

A comprehensive evaluation was performed on all patients, including age, anthropometric data (body mass index (BMI) and circumferences of neck, waist, and hip), sleep-related symptoms, relevant medical history (including comorbidities), and histories of smoking, alcohol consumption, and substance abuse. Furthermore, attended overnight polysomnography (PSG) studies were performed. Participants’ subjective daytime sleepiness levels were determined with the Epworth Sleepiness Scale (ESS).

#### Epworth Sleepiness Scale

The ESS is presently the most frequently employed self-reported measure of daytime sleepiness in clinical practice. This self-administered assessment consists of a concise eight-question questionnaire. The tool measures how likely someone is to fall asleep in eight typical situations. A score under 10 is within the normal range. Subjective daytime sleepiness increases proportionally with scores ranging from 10 to 24. An ESS score exceeding 10 points (ESS ≥ 11) suggests excessive daytime sleepiness (EDS) [26].

### 2.3. Polysomnography

Each patient received a single-night, full diagnostic PSG study using the Alice 5 Diagnostics System (Respironics, Murrysville, PA, USA), adhering to standard protocols and including the monitoring of the electroencephalogram (EEG; using 3 electroencephalogram derivations: frontal, central, and occipital), electro-oculogram, electromyogram, flow (by oronasal thermistor and nasal air pressure transducer), thoracic and abdominal respiratory effort (by respiratory inductance plethysmography), pulse oximetry (SpO_2_), and body position. A microphone positioned on the anterior neck documented instances of snoring. Apnea and hypopnea were defined according to the American Academy of Sleep Medicine’s standard criteria including the hypopnea rule, which requires ≥3% desaturation or EEG-based arousal [27]. The analysis included the following parameters: total sleep time (TST), sleep efficiency [SE (%), percentage of total time in bed actually spent in sleep], wakefulness after sleep onset (WASO, the amount of wake periods after sleep onset), arousal index (AI), apnea–hypopnea index (AHI), oxygen desaturation index (ODI), resting room air pulse oximetry (SpO_2_), and sleep time with oxygen saturation < 90% (TST90). The AHI, computed as the hourly frequency of apnea and hypopnea events during sleep, served as the diagnostic criterion for OSA and its severity assessment. The severity of OSA was categorized according to the AHI: mild (5 to <15 events/h), moderate (15 to <30 events/h), and severe (≥30 events/h). Only participants with an AHI of at least 5 events per hour were included in our study.

### 2.4. Statistical Analysis

For continuous variables, means and standard deviations (mean ± SD) are reported if they are normally distributed; otherwise, medians and interquartile ranges (25th–75th percentile) are given. The qualitative variables are represented by absolute numbers and percentages. Normally distributed variables were analyzed with ANOVA to determine differences among mild-, moderate-, and severe-OSA patient groups. Significant ANOVA results were followed by pairwise comparisons using the Tukey–Kramer post hoc test. For non-normally distributed variables, the Kruskal–Wallis test was used for comparing the three groups. Following a significant Kruskal–Wallis test, Dunn’s pairwise comparisons were performed for the three group pairs, adjusting for multiple testing with the Bonferroni correction. Chi-square analysis was used to compare the three groups on categorical variables. Each of the three OSA severity groups was examined for association with prevalent EDS. A logistic regression analysis was conducted to determine the variables associated with EDS, controlling for age, gender, obesity indicators (BMI, waist-to-hip ratio, and neck circumference), smoking status, and comorbidities. An assessment of multicollinearity among predictors was conducted by using tolerance and the variance inflation factor (VIF) to maintain acceptable levels of collinearity. Age was categorized into groups of 18–59 and ≥60 years; the BMI was categorized as obese (BMI ≥ 30 kg/m^2^) and non-obese (BMI < 30 kg/m^2^); and EDS was categorized as sleepy (ESS >10) and non-sleepy (ESS ≤ 10). Statistical significance was established for *p*-values less than 0.05. Data analysis was conducted by using SPSS version 25 (SPSS Inc., Chicago, IL, USA).

## 3. Results

### 3.1. Patient Characteristics

The descriptive characteristics of the study population and the distribution of covariates are shown in Table 1. Overall, participants were predominantly males (79%), middle-aged (50 ± 14 years), and obese (BMI of 33 ± 7 kg/m^2^). The majority of the participants (89%) had a spouse and had a higher level of education (68%). Individuals in the severe-OSA group were older and more obese, were more frequently males, and had higher prevalence of hypertension and hyperlipidemia compared with mild- and moderate-OSA patients.

### 3.2. General Characteristics of the EDS Phenotype

Almost half of the population presented EDS, with significant differences between the three OSA severity groups (Table 1). The severe-OSA group exhibited the highest EDS (57 vs. 46 vs. 38%, *p* < 0.001). In the whole population, the sleepy phenotype was on average more often found in those who were males (82 vs. 75%, *p* = 0.011), younger than 60 years (77 vs. 70%, *p* = 0.011), and obese (67 vs. 59%, *p* = 0.006) compared with the non-sleepy phenotype. In the moderate-OSA group, the sleepy phenotype was more often found in those younger than 60 years (78 vs. 67%, *p* = 0.02), and in the severe-OSA group, the sleepy phenotype was more often found in those who were male (85 vs. 79%, *p* = 0.042) and younger than 60 years (73 vs. 65%, *p* = 0.038).

### 3.3. The Effect of PSG Parameters on EDS

**Figure 2:**
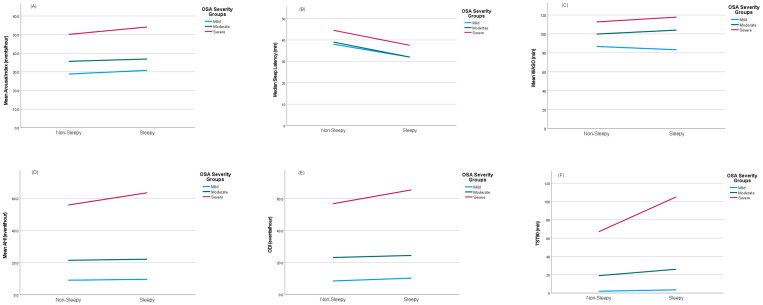
Comparison of arousal index (**A**), sleep latency (**B**), WASO (**C**), AHI (**D**), ODI (**E**), and TST90 (**F**) between sleepy and non-sleepy groups within OSA severity groups.

Overall, the participants had moderate-to-severe OSA, with a median AHΙ of 31 (19, 61) events/hour. All patients and the patients in the three OSA severity groups were further divided into two categories based on their level of daytime sleepiness (Supplementary Table S1, Table 2, Figure 2): those who experienced EDS (ESS > 10) and those who did not (ESS ≤ 10). In all patients, SWS sleep (7 vs. 8% TST, *p* = 0.02) and sleep latency were lower (35 vs. 41, *p* < 0.001), whereas the arousal index (47 vs. 41, *p* < 0.001), WASO (110 vs. 103, *p* = 0.004), and NREM sleep (91 vs. 89, *p* = 0.03) were higher. The indices of OSA severity (AHI, ODI, mean and lowest SpO_2_, and TST90, all *p* < 0.001) were worse in sleepy OSA patients compared with non-sleepy ones. Mild-OSA sleepy patients exhibited higher WASO (92 vs. 88, *p* = 0.03) compared with non-sleepy patients. Decreased sleep latency (32 vs. 39, *p* = 0.03) was noted in moderate-OSA sleepy patients. Statistically significant differences were observed in the mean (93 vs. 94%, *p* = 0.005) and lowest SpO_2_ (84 vs. 84%, *p* = 0.01) and in TST90 (26 vs. 19, *p* = 0.001); however, these differences lacked clinical significance. In the severe-OSA sleepy subgroup, SWS sleep (7 vs. 8% TST, *p* = 0.001) and sleep latency were lower (38 vs. 45, *p* < 0.001), whereas the arousal index (54 vs. 50, *p* = 0.001) was higher. Additionally, the indices of OSA severity (AHI, ODI, mean and lowest SpO_2_, and TST90, all *p* < 0.001) were worse in severe-OSA sleepy patients.

Table 3 summarizes the results of a multiple stepwise logistic regression analysis exploring the association between EDS and various independent factors, considering the entire patient population and the OSA severity classifications separately. After adjustments for age, gender, BMI, smoking status, and comorbidities, we found that for the entire population, male sex, younger age (<60 years), obesity (BMI ≥ 30kg/m^2^), the presence of depression, WASO, lower sleep efficiency, the arousal index, and all indices of OSA severity were significantly associated with the presence of EDS. An arousal index exceeding 30 was the most significant predictor, demonstrating a two-fold increased likelihood of developing EDS.

It is also noteworthy that the arousal index consistently showed a strong correlation with EDS across all OSA severity groups; this was particularly true for the mild-OSA group, in which it was the only significant predictor. Male gender, obesity, and the presence of depression, along with WASO and lower SWS, remained significant predictors of EDS only in the severe-OSA group. Patients with moderate-to-severe OSA who were younger and exhibited shorter sleep latencies were more likely to experience EDS. While the AHI significantly predicted EDS only in severe-OSA cases, the ODI, the mean and lowest SpO2, and TST90 were predictive of EDS in both moderate- and severe-OSA groups.

## 4. Discussion

This study explores differences in PSG characteristics between sleepy and non-sleepy patients in a large cohort of treatment-naïve patients with different levels of OSA severity. Our findings indicate the presence of distinct sleepy phenotypes, varying according to the severity of OSA. More specifically, the mild-OSA sleepy patient was characterized by a higher arousal index, whereas the moderate-OSA sleepy patient was younger, with shorter sleep latency and increased indices of OSA severity, excluding the AHI in addition to an increased arousal index. Patients in the severe-OSA sleepy group tended to be younger, males, obese, and diagnosed with depression (on medication). Those patients also exhibited decreased SWS and sleep latency, increased WASO, an arousal index, and an increase in all indices of OSA severity.

One major finding of our study is that an increased arousal index (≥30) was associated with a 2.4 greater likelihood of subjective EDS regardless of OSA severity. This finding is in accordance with previous studies [18,20,22,28,29,30]; however, other studies [16,30,31,32] have failed to establish a statistically significant association between the arousal index and sleepiness in OSA. Evidence suggests that recurrent cortical arousals indicate physiological stress and impair the restorative function of sleep, resulting in daytime sleepiness and cognitive impairment [33]. Although they present as promising predictors of EDS, EEG-based arousals are scored less reliably compared with respiratory events and are influenced by sleep stages [34,35]. Additionally, their association with sympathetic activation is unclear [36], suggesting the need for alternative methods of measuring arousal during sleep. The addition of “arousal intensity” to arousal frequency has been recommended as a potential way to improve their predictive value [37]. Notably, a recent study explored markers of arousal intensity, in addition to the arousal index [30]. The authors suggested that while the number of arousals may be similar between sleepy and not sleepy patients, sleepy phenotypes might experience more disruptive or severe arousals accompanied by sympathetic nervous system activation, potentially resulting in heightened daytime sleepiness [30]. Additionally, it has been shown that cortical arousals with a duration greater than 15 s exhibit a more robust association with ESS scores than shorter cortical arousals (3–15 s) [38]. In light of these findings, we consider that arousal events during sleep deserve increased attention in polysomnographic scoring, as they reliably reflect daytime sleepiness and sleep quality. However, additional research is essential to gaining a more comprehensive understanding of this association.

We also explored other markers of sleep architecture, and we found that in moderate-to-severe-OSA patients, the sleepy phenotype, compared with the not sleepy phenotype, had shorter sleep latency, spent more time awake after sleep onset and had less SWS in severe OSA, in accordance with previous studies [20,22,39]. New research using innovative methods to assess sleep architecture reinforces the association between EDS and reduced SWS [40]. In this study, OSA patients with severe objective EDS, identified by the Multiple Sleep Latency Test, exhibited lower N3 sleep continuity and duration [40]. Our findings, however, contradict earlier research in OSA patients that did not link EDS to alterations in sleep architecture [16,23,30,31].

Another important finding of our study is that the EDS in our cohort was associated with more severe hypoxemia, as indicated by increased ODI and TST90, as well as lower mean and lowest SpO_2_, in moderate-to-severe-OSA patients. These findings are in line with prior research [6,16,17,29,30,31], which consistently found significantly worse hypoxemia in OSA patients with EDS. While several mechanisms have been proposed for how hypoxemia induces sleepiness in OSA, including inflammation, oxidative injury, neuronal damage, and cell loss in wake-promoting regions of the brain [41,42,43], the exact mechanisms that lead to EDS in OSA are still unclear. Importantly, our study found that the association between OSA severity as traditionally measured by the AHI and EDS was only evident in patients with severe OSA. This is not unexpected, given that the relationship between the AHI and EDS is uncertain and even poor [3,15,16,17,18,20,22]. In this context, models utilizing hypoxemia metrics as predictors of EDS could be considered preferable to those relying on the AHI. The OSA-specific hypoxic burden, measuring the frequency, depth, and duration of desaturation during respiratory events, could show promise due to its significant associations with various negative health outcomes [44].

While most research has focused on moderate and severe OSA, this study delves deeper by exploring predictors of EDS separately for each severity level, including mild OSA. Our study shows a high rate of EDS among patients with mild OSA, aligning with results from previous studies [45,46,47]. The EDS experienced by patients with mild OSA may be a result of an increased arousal index, as observed in our study. This suggests that these patients could represent a unique subgroup within the OSA population, and the arousal index might be a better marker for their sleepiness than traditional OSA severity measures. However, it is important to consider that there may be additional unmeasured factors contributing to EDS in these patients, such as habitual sleep duration and medications other than benzodiazepines that could influence EDS.

Demographic characteristics that may be associated with EDS in OSA patients have also been described in our study. Younger age has been associated with EDS, particularly in moderate- and severe-OSA patients [11], and this is consistent with previous studies [20,48]. However, the influence of aging on the self-reported daytime sleepiness in OSA [49] should be taken into account, particularly when the ESS, one of the most common screening instruments for assessing sleepiness, is used [26]. The ESS evaluates sleepiness by questioning the tendency to fall asleep in eight common everyday scenarios [26], where older individuals may not engage in all of them [50]. Consequently, lower ESS score and EDS prevalence are expected in this age group. Our findings also indicate a higher prevalence of EDS in males, especially in the group with severe OSA. It appears that the ESS is more likely to identify sleepiness in men than in women [13,51]. This might be because women answer questions about sleepiness differently and might not experience the typical symptoms of OSA [52,53]. The higher BMI in severe-OSA sleepy patients noted in our study has also been independently associated with sleepiness in OSA patients in previous studies [22,54].

Our study expands upon existing knowledge of OSA by identifying factors that predict EDS in clinical populations with OSA. Examining the PSG factors contributing to the sleepiness phenotype is a critical step in understanding the association between OSA’s pathophysiology and EDS experienced by patients. The identification of these distinctive PSG findings can facilitate clinicians in providing a more precise assessment of EDS in these patients, thus promoting effective individualized management and mitigating potential health complications.

One strength of this study is the large, well-characterized sample of treatment naïve patients with OSA, with detailed characterization of PSG parameters. We also excluded patients with major comorbidities and other sleep disorders that could influence sleep architecture, and we explored how the observed relationships between the sleepiness phenotypes may differ based on clinical and PSG characteristics. Furthermore, we had data on depression available; therefore, we found that this variable might influence our sleepiness phenotype. There are also limitations. The patients with OSA were recruited from a sleep center and do not necessarily represent patients with OSA found in the general population. Moreover, as males were overrepresented in our population, our findings may not be generalizable to females. Another limitation is that the ESS was used for EDS evaluation, which is less reliable than an objective assessment of sleepiness, such as the Multiple Sleep Latency Test, and may underestimate sleepiness severity in older subjects [50]. An objective assessment of sleepiness is highly warranted in future studies to validate these results. Further research is also needed to establish causal relationships between PSG parameters and EDS; investigate these differences in more diverse populations, with a particular focus on including a greater number of female participants; and explore additional contributing factors, such as habitual sleep time and medications for EDS in OSA patients.

## 5. Conclusions

Our results suggest that male sex, younger age, obesity, the presence of depression, parameters of sleep architecture, and indices of OSA severity may account for the presence or absence of EDS in OSA patients. Despite identifying distinct sleepy phenotypes based on OSA severity, the arousal index consistently showed a strong correlation with EDS across all OSA severity groups. The next important step is to explore the role and mechanisms of these factors in causing sleepiness and find valid sleepiness measures that can accurately predict health risks in OSA patients.

## Figures and Tables

**Table 1:** Clinical characteristics of patients according to OSA severity.

	Total Population N = 1307	Total Population According to AHI			
		Mild-OSA Patients (AHI 5 to <15/h) N = 236	Moderate-OSA Patients (AHI 15 to <30/h) N = 367	Severe-OSA Patients (AHI ≥ 30/h) N = 704	*p*-Value Across All
**Demographics**					
Sex, males (%)	1027 (79%)	172 (73%)	270 (74%)	585 (83%) **^, #^	<0.001
Age, years	50 ± 14	44 ± 13.0	51 ± 14 *	52 ± 14 **	<0.001
Age ≥ 60 years (%)	345 (27%)	27 (12%)	104 (28%) *	214 (30%) **	<0.001
BMI (kg/m^2^)	33 ± 7	29 ± 5	31 ± 6*	35 ± 7 **^, #^	<0.001
BMI ≥ 30, n (%)	822 (63%)	87 (37%)	187 (51%) *	548 (78%) ^#,^ **	<0.001
NC (cm)	42 ± 5	39 ± 4	41 ± 4 *	43 ± 5 **^, #^	<0.001
WC (cm)	111 ± 16	101 ± 12	107 ± 12 *	117 ± 16 **^, #^	<0.001
HC (cm)	112 ± 15	105 ± 12	109 ± 12 *	117 ± 15 **^, #^	<0.001
Waist-to-hip ratio	0.99 ± 0.07	0.96 ± 0.08	0.99 ± 0.06 *	1.00 ± 0.07 **^, #^	<0.001
**Education Level**					
Primary level	61 (5%)	7 (3%)	19 (5%)	35 (5%)	
Secondary level	356 (27%)	52 (22%)	93 (25%)	211 (30%)	
Higher level	890 (68%)	177 (75%)	255 (69%)	458 (65%)	0.072
**Married/with partner (%)**	1160 (89%)	186 (78%)	328 (89%)	647 (92%)	<0.001
**Smoking status**					
Never, n (%)	501 (38%)	90 (38%)	152 (41%)	259 (37%)	
Current/former, n (%)	806 (62%)	146 (62%)	215 (59%)	445 (63%)	0.461
**Daytime sleepiness**					
ESS	10 ± 5	8 ± 5	9 ± 5	11 ± 6 **^, #^	<0.001
ESS > 10 (%)	644 (49%)	88 (38%)	165 (46%) *	391 (57%) **^, #^	<0.001
**Comorbidities (%)**					
Arterial hypertension	435 (33%)	29 (12%)	122 (33%) *	284 (40%) **^, #^	<0.001
Hyperlipidemia	469 (36%)	62 (26%)	136 (37%) *	271 (39%) **	0.003
Depression (on medications)	119 (9%)	18 (8%)	32 (9%)	69 (10%)	0.585

Data are presented as N (%) for categorical variables and mean values  ±  SDs or medians (25th–75th percentiles) for continuous variables. AHI: apnea–hypopnea index; BMI: body mass index; ESS: Epworth Sleepiness Scale; HC: hip circumference; NC: neck circumference; OSA: obstructive sleep apnea; WC: waist circumference. * *p* < 0.05, mild-OSA vs. moderate-OSA groups; ** *p* < 0.05, mild- vs. severe-OSA groups; # *p* < 0.05, moderate- vs. severe-OSA groups.

**Table 2:** Comparison of study subgroups with or without EDS within OSA severity groups.

	Mild OSA			Moderate OSA			Severe OSA		
	ESS ≤ 10	ESS > 10	*p*-Value	ESS ≤ 10	ESS > 10	*p*-Value	ESS ≤ 10	ESS > 10	*p*-Value
**TST (min)**	292 ± 49	296 ± 48	0.66	275 ± 56	281 ± 52	0.28	262 ± 61	261 ± 54	0.86
**Sleep efficiency (%)**	70 ± 10	72 ± 10	0.35	66 ± 12	67 ± 11	0.33	62 ± 13	63 ± 11	0.42
**NREM (%TST)**	89 ± 8	89 ± 4	0.94	88 ± 7	90 ± 7	0.86	91 ± 6	90 ± 3	0.86
**SWS (%TST)**	8 ± 4	8 ± 3	0.70	8 ± 3	8 ± 4	0.93	8 ± 4	7 ± 3	0.001 *
**REM (%TST)**	11 ± 4	12 ± 4	0.62	10 ± 4	11 ± 4	0.24	10 ± 8	9 ± 4	0.51
**Arousal index**	30 ± 9	31 ± 7	0.65	37 ± 8	37 ± 7	0.54	50 ± 14	54 ± 13	0.001 *
**Sleep latency (min)**	38 (26, 58)	32 (21, 53)	0.24	39 (25, 61)	32 (22, 52)	0.03 *	45 (29, 75)	38 (24, 59)	<0.001 *
**WASO (min)**	88 ± 33	92 ± 33	0.03 *	106 ± 38	107 ± 38	0.63	118 ± 39	122 ± 40	0.19
**AHI**	9 ± 3	10 ± 3	0.06	21 ± 4	22 ± 4	0.13	55 ± 20	63 ± 23	<0.001 *
**AHI REM**	12 ± 7	13 ± 7	0.52	29 ± 13	30 ± 12	0.40	59 ± 23	64 ± 23	0.01 *
**ODI**	8 ± 3	10 ± 9	0.11	24 ± 9	24 ± 6	0.52	57 ± 22	65 ± 24	<0.001 *
**Mean SpO_2_ (%)**	95 ± 1	95 ± 1	0.23	94 ± 2	93 ± 1	0.005 *	91 ± 2	90 ± 3	<0.001 *
**Lowest SpO_2_ (%)**	88 ± 3	88 ± 4	0.39	84 ± 3	83 ± 4	0.01 *	78 ± 7	74 ± 8	<0.001 *
**TST90 (min)**	2 (0, 7)	4 (1, 9)	0.12	19 (10, 33)	26 (14, 43)	0.001 *	67 (38, 108)	105 (59, 171)	<0.001 *
**TST90 (%)**	0.8 (0, 2.3)	1.3 (0.3, 1.3)	0.11	7.2 (3.6, 12.0)	9.4 (4.7, 15.6)	0.001 *	25.9 (13.8, 46.0)	42.7 (21.8, 71.4)	<0.001 *

AHI: apnea–hypopnea index; AI: arousal index; ESS: Epworth Sleepiness Scale; ODI: oxygen desaturation index; OSA: obstructive sleep apnea; SE: sleep efficiency; SpO_2_: resting room air pulse oximetry; TST: total sleep time; TST90: sleep time with oxygen saturation < 90%; WASO, wakefulness after sleep onset. * *p*-Value < 0.05.

**Table 3:** Multiple stepwise logistic regression analysis of relationship between EDS and various independent variables in OSA severity groups.

	All OSA Patients		Mild-OSA Patients		Moderate-OSA Patients		Severe-OSA Patients	
	OR (95%CI)	*p*-Value	OR (95%CI)	*p*-Value	OR (95%CI)	*p*-Value	OR (95%CI)	*p*-Value
**Males vs. females**	1.499 (1.129–1.991)	0.005 *	0.751 (0.393–1.436)	0.386	1.399 (0.821–2.386)	0.217	1.797 (1.160–2.784)	0.009 *
**Age ≤ 60**	1.418 (1.080–1.864)	0.012 *	0.433 (0.149–1.254)	0.123	1.802 (1.079–3.012)	0.025 *	1.359 (1.053–1.938)	0.041 *
**BMI ≥ 30**	1.351 (1.069–1.707)	0.012 *	0.887 (0485–1.624)	0.699	0.931 (0.588–1.474)	0.759	1.025 (1.002–1.049)	0.035 *
**Depression**	1.688 (1.128–2.527)	0.011 *	1.033 (0.367–2.905)	0.952	1.692 (0.785–3.648)	0.180	1.829 (1.051–3.184)	0.033 *
**Sleep efficiency (%)**	1.003 (0.993–1.013)	0.582	1.028 (0.996–1.060)	0.090	1.015 (0.994–1.037)	0.161	1.015 (0.999–1.031)	0.063
**WASO**	1.004 (1.001–1.007)	0.015 *	0.997 (0.989–1.006)	0.543	1.003 (0.996–1.009)	0.418	1.002 (0.998–1.007)	0.049 *
**Sleep latency**	0.995 (0.991-.998)	0.003 *	1.000 (0.990–1.009)	0.969	0.993 (0.986–1.000)	0.038 *	0.989 (0.983–0.944)	<0.001 *
**NREM (%TST)**	1.000 (0.999–1.002)	0.633	1.011 (0.969–1.055)	0.601	0.966 (0.963–1.031)	0.841	1.000 (0.999–1.002)	0.633
**SWS (%TST)**	0.960 (0.928–0.993)	0.018 *	0.964 (0.870–1.069)	0.487	1.050 (0.953–1.156)	0.325	0.922 (0.874–0.972)	0.003 *
**REM (%TST)**	0.995 (0.973–1.018)	0.658	1.005 (0.942–1.071)	0.889	1.080 (0.976–1.195)	0.139	0.986 (0.954–1.020)	0.418
**Arousal index**	1.026 (1.017–1.035)	<0.001 *	1.043 (1.003–1.085)	0.036 *	1.026 (1.007–1.055)	0.038 *	1.020 (1.008–1.033)	0.002 *
**Arousal** **index ≥ 30**	2.398 (1.702–3.380)	<0.001 *	2.412 (1.373–4.239)	0.002 *	2.229 (1.236–4.020)	0.008 *	2.001 (0.326–12.267)	0.033 *
**AHI**	1.014 (1.009–1.018)	<0.001 *	1.098 (0.987–1.222)	0.086	1.065 (0.997–1.138)	0.062	1.016 (1.007–1.024)	<0.001 *
**AHI REM**	1.011 (1.006–1.015)	<0.001 *	1.008 (0.968–1.049)	0.699	1.013 (0.994–1.033)	0.190	1.008 (1.001–1.016)	0.032 *
**ODI**	1.014 (1.010–1.019)	<0.001 *	1.050 (0.971–1.136)	0.221	1.046 (1.011–1.082)	0.009 *	1.016 (1.008–1.024)	<0.001 *
**Mean SaO_2_**	0.817 (0.775–0.861)	<0.001 *	0.828 (0.632–1.085)	0.171	0.712 (0.602–0.842)	<0.001 *	0.794 (0.726–0.870)	<0.001 *
**Lowest SaO_2_**	0.936 (0.920–0.953)	<0.001 *	0.948 (0.844–1.065)	0.368	0.890 (0.831–0.954)	0.001 *	0.926 (0.899–0.952)	<0.001 *
**TST90 (%)**	1.019 (1.014–1.024)	<0.001 *	1.051 (0.960–1.150)	0.278	1.022 (1.004–1.040)	0.016 *	1.018 (1.012–1.024)	<0.001 *

All models were adjusted for the participants’ age, sex, obesity, smoking status, and comorbidities. * *p*-Value < 0.05.

## Data Availability

The data presented in this study are available upon request from the corresponding author. The data are not publicly available due to privacy restrictions.

## Supplementary Materials

The following supporting information can be downloaded at: https://www.mdpi.com/article/10.3390/healthcare13050478/s1, Supplementary Table S1: Comparison of PSG characteristics in total population based on their level of daytime sleepiness.
