# Supplementary material for: Polysomnography Differences Between Sleepy and Non-Sleepy Obstructive Sleep Apnea (OSA) Patients
Source: Healthcare (Basel). 2025 Feb 22;13(5):478. doi: 10.3390/healthcare13050478 (PMC11898945; doi:10.3390/healthcare13050478)
Supplement: Supplementary file 1 [file healthcare-13-00478-s001.zip › healthcare-3423333-supplementary.pdf]

Supplementary Table S1. Comparison of PSG characteristics in total population based on their level of daytime sleepiness.

	Total population		
	**ESS≤10**	**ESS>10**	**p-Value**
**TST (min)**	273 ± 58	271 ± 54	0.54
**Sleep Efficiency (%)**	65 ± 12	65 ± 11	0.99
**NREM (%TST)**	89 ± 12	91± 6	0.03*
**SWS (%TST)**	8 ± 4	7 ± 4	0.02*
**REM (%TST)**	9 ± 4	0 ± 3	0.89
**Arousal Index**	41 ± 14	47 ± 15	<0.001*
**Sleep Latency (min)**	41 (27, 65)	35 (23, 56)	<0.001*
**WASO (min)**	103 ± 41	110 ± 42	0.004*
**AHI**	35 ± 25	46 ± 29	<0.001*
**AHI REM**	40 ± 27	48 ± 28	<0.001*
**ODI**	36 ± 26	47 ± 30	<0.001*
**Mean SpO_2 (%)**	93 ± 2	92 ± 3	<0.001*
**Lowest SpO_2 (%)**	82 ± 7	78 ± 8	<0.001*
**TST90 (min)**	28 (8, 69)	58 (20, 132)	<0.001*
**TST90 (%)**	10 (3, 27)	21 (7, 53)	<0.001*

AHI: apnoea-–hypopnoea index, AI: arousal index, ESS: Epworth Sleepiness Scale, ODI: oxygen desaturation index, OSA: Obstructive Sleep Apnoea, SE: Sleep Efficiency, SpO_2: resting room air pulse oximetry, TST: total sleep time, TST90: sleep time with oxygen saturation < 90%: WASO wakefulness e after sleep onset. * p-value <0.05.
